# Wheel running improves fasting‐induced AMPK signaling in skeletal muscle from tumor‐bearing mice

**DOI:** 10.14814/phy2.14924

**Published:** 2021-07-16

**Authors:** Dennis K. Fix, Brittany R. Counts, Ashley J. Smuder, Mark A. Sarzynski, Ho‐Jin Koh, James A. Carson

**Affiliations:** ^1^ Department of Exercise Science Arnold School of Public Health University of South Carolina Columbia SC USA; ^2^ Integrative Muscle Biology Laboratory Division of Rehabilitation Sciences College of Health Professions University of Tennessee Health Science Center Memphis TN USA; ^3^ Department of Applied Physiology & Kinesiology College of Health & Human Performance University of Florida Gainesville FL USA

**Keywords:** cachexia, muscle wasting, physical activity, protein breakdown

## Abstract

Disruptions to muscle protein turnover and metabolic regulation contribute to muscle wasting during the progression of cancer cachexia. The initiation of cachexia is also associated with decreased physical activity. While chronic muscle AMPK activation occurs during cachexia progression in Apc*^Min^* ^/+^ (MIN) mice, a preclinical cachexia model, the understanding of muscle AMPK’s role during cachexia initiation is incomplete. Therefore, we examined if voluntary wheel exercise could improve skeletal muscle AMPK signaling in pre‐cachectic MIN mice. Next, we examined muscle AMPK’s role in aberrant catabolic signaling in response to a 12‐h fast in mice initiating cachexia. Male C57BL/6 (B6: *N* = 26) and MIN (*N* = 29) mice were subjected to ad libitum feeding, 12‐h fast, or 4 wks. of wheel access and then a 12‐h fast during the initiation of cachexia. Male tamoxifen‐inducible skeletal muscle AMPKα ***^1^*** α ***^2^*** (KO) knockout mice crossed with Apc*^Min^* ^/+^ and floxed controls were examined (WT: *N* = 8, KO: *N* = 8, MIN: *N* = 10, MIN KO: *N* = 6). Male mice underwent a 12‐h fast and the gastrocnemius muscle was analyzed. MIN gastrocnemius mass was reduced compared to B6 mice. A 12‐h fast induced MIN muscle AMPK^T172^, FOXO^S413^, and ULK‐1^S555^ phosphorylation compared to B6. Wheel running attenuated these inductions. A 12‐h fast induced MIN muscle MuRF‐1 protein expression compared to B6 and was suppressed by wheel running. Additionally, fasting induced muscle autophagy signaling and disrupted mitochondrial quality protein expression in the MIN, which was prevented in the MIN KO. We provide evidence that increased skeletal muscle AMPK sensitivity to a 12‐h fast is an adverse event in pre‐cachectic MIN mice, and exercise can improve this regulation.

## Graphical abstract

These results provide evidence that MIN mouse muscle AMPK signaling has greater sensitivity to a 12hr fast than B6 mice, and wheel running is sufficient to lower this fasting response.

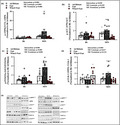

## 1 INTRODUCTION

Cancer‐induced cachexia is a debilitating wasting condition resulting in 40% of all cancer‐related deaths (Argiles et al., 2010). The loss of skeletal muscle mass and function is a hallmark of cancer cachexia, and this loss is directly related to increased morbidity and mortality (Baracos, 2011; Baracos et al., 2018). Skeletal muscle mass regulation involves a balance between the rates of protein synthesis and degradation (Maddocks et al., 2011; Marimuthu et al., 2011). Disrupted muscle protein turnover is a foundation of cancer‐induced wasting (Aversa et al., 2016; Baracos, 2000). The disruption of muscle protein turnover with cancer cachexia corresponds to the chronic activation of adenosine monophosphate protein kinase (AMPK), which has been widely observed in preclinical cachexia models (Liu et al., 2019; Puppa et al., 2014; Segatto et al., 2017; White, Puppa, Gao, et al., 2013) and observed in human cachectic patients (Segatto et al., 2017). When activated, AMPK will phosphorylate tuberous sclerosis protein 2 (TSC2) and Raptor, inhibiting mTORC1 activity (Gwinn et al., 2008). AMPK regulates skeletal muscle proteasomal degradation by upregulating muscle‐specific E3 ligases Atrogin‐1/MAFbx and MuRF‐1 controlled upstream by FOXO3a (Greer et al., 2007). AMPK also regulates autophagic–lysosomal proteolysis through AMPK’s activation of ULK^S555^ (Lira et al., 2013) and mTORC1 inhibition. Additionally, ULK1 phosphorylation ^S757^ by mTORC1 suppresses the phosphorylation of ULK1^S555,^ thereby inhibiting AMPK. While there has been extensive investigation of skeletal muscle protein turnover during the initiation and progression of cachexia (Huot et al., 2020; Liu et al., 2018; Pigna et al., 2016; Talbert et al., 2019), AMPK’s role in these processes is far less understood.

Skeletal muscle protein synthesis and degradation capacity oscillate throughout the day in response to feeding, fasting. and physical activity (Burd et al., 2009; Phillips, 2014), and cancer negatively impacts this regulation (Dijk et al., 2015; Hardee et al., 2018a; Phillips et al., 2013; Puppa et al., 2014; Counts et al., 2020). Adequate nutrient availability is critical for the net gain and loss of skeletal muscle protein (Burd et al., 2009) and AMPK is a vital nutrient and energy sensor for maintaining skeletal muscle metabolic homeostasis (Koh et al., 2008a). Notably, chronic AMPK activity in cachectic mice has not been linked to decreased food intake (Hardee, Counts, et al., 2018; Puppa et al., 2014; Counts et al., 2020). Independent of cancer, muscle AMPK activation by a prolonged fast or starvation has been widely investigated (Bujak et al., 2015). For example, fasting‐induced starvation (>24 h) is known to induce AMPK and associated signaling (Bujak et al., 2015; Castets et al., 2013), while short‐term fasting <12 h is commonly used throughout the literature to standardize testing to reduce variability (Jensen et al., 2013).

Cancer cachexia exhibits reduced volitional activity, whole body weakness, and fatigue in cancer patients and preclinical models (Baltgalvis et al., 2010; Murphy et al., 2012; Toth et al., 2016). During the initiation of cachexia, tumor‐bearing mice exhibit reduced overall daily activity and increased muscle fatigue (Baltgalvis et al., 2010; Murphy et al., 2012; VanderVeen et al., 2018) and physical inactivity increases during the progression of cancer cachexia (Murphy et al., 2020). Importantly, increased activity in tumor‐bearing mice, before indices of cachexia, is sufficient to maintain muscle mass (Murphy et al., 2020; Puppa et al., 2012; Vanderveen et al., 2020), supporting the therapeutic potential of increased activity on preventing or delaying disease onset. Physical activity has been implicated in attenuating muscle mass loss during a severe energy deficit (Martin‐Rincon et al., 2019), further emphasizing exercise's potential therapeutic role to offset the adverse events of multiple metabolic stress conditions. Exercise training is a known inducer of muscle anabolic signaling through mTORC1 (Burd et al., 2009) and reduces chronic inflammation (Beavers et al., 2010). Importantly, an acute bout of exercise induces short‐term activation of AMPK that can initiate glucose uptake, fatty acid oxidation, and mitochondrial biogenesis (Egan & Zierath, 2013). AMPK phosphorylation of ULK has been implicated as a necessary exercise induced adaptation, highlighting the importance of AMPK in the exercise response (Laker et al., 2017). Additionally, it well known that cancer cachexia induces the chronic activation of AMPK in skeletal muscle; however, regular exercise can prevent the chronic induction of muscle AMPK in tumor‐bearing mice (Puppa et al., 2012), further underscoring exercise's effect in preventing indices of cachexia (Coletti et al., 2016; Mehl et al., 2005; Pigna et al., 2016; Vanderveen et al., 2020; Jee et al., 2016). Most preclinical models adding a physical activity or exercise treatment before either the rapid tumor growth stage or cachexia onset can attenuate or prevent wasting (Gould et al., 2013; Hardee et al., 2019). Furthermore, impaired systemic metabolism (Das et al., 2011; Han et al., 2020; Kir et al., 2014) and muscle metabolic dysfunction (Brown et al., 2017; Han et al., 2020) have been reported before cachexia onset and are potential drivers of accelerated protein turnover during cachexia's progression(VanderVeen et al., 2017). However, the effect of exercise on the feeding and fasting metabolic regulation during cachexia initiation is uncertain.

AMPK’s regulatory role in muscle metabolism has been extensively researched (Jeon, 2016; Kim et al., 2016; Kjobsted et al., 2018; Steinberg & Jorgensen, 2007). Several studies have investigated AMPK loss in heart and skeletal muscle (Kjobsted et al., 2018; Viollet et al., 2009), which exhibit decreased functional outcomes in run time to fatigue, daily wheel distance, or cage activity (Morissette et al., 2014; Zwetsloot et al., 2008; Maarbjerg et al., 2009). More specifically, in skeletal muscle, AMPK α2 muscle‐specific knockout (Miura et al., 2009) and AMPK α2 kinase dead (Moller et al., 2016) decreased running capacity, AMPK β1/β2 muscle loss decreased muscle fiber size and capillary density (Thomas et al., 2014), and AMPK γ3 loss decreased glycogen content (Canto et al., 2010). These studies highlight the importance of AMPK in skeletal muscle under normal physiological conditions. Yet, the effect of muscle AMPK loss with chronic disease is less understood and is an important consideration since disease conditions such as cachexia exhibit chronically elevated AMPK signaling. To date, there are a limited number of studies that have examined the impact of cancer cachexia progression on AMPK regulation in skeletal muscle. Briefly, AMPK activators, such as AICAR and Metformin administered before cachexia initiation, have been examined with some preclinical models of cancer cachexia. AICAR administration can prevent muscle mass loss in tumor‐bearing mice (Hall et al., 2018; Pigna et al., 2016) and lower E3 ligase gene expression and autophagy indices (Pigna et al., 2016). Metformin can also prevent indices of cachexia (Hall et al., 2018; Oliveira & Gomes‐Marcondes, 2016). Despite these facts, there is a limited understanding of AMPK’s regulation in skeletal muscle during cancer cachexia progression.

The MIN mouse is an established preclinical model of cancer‐induced cachexia (Baltgalvis et al., 2010). In cachectic tumor‐bearing mice, skeletal muscle AMPK signaling is induced following a relatively short fasting (5 h) and suggests muscle metabolic dysfunction (Penna et al., 2010; White et al., 2011; White, Puppa, Gao, et al., 2013). This induction of muscle AMPK coincides with mTORC1 suppression (Hardee, Fix, et al., 2018; Hardee et al., 2016; White, Puppa, Gao, et al., 2013). While cachectic muscle exhibits suppressed anabolic signaling by chronic AMPK activity (White, Puppa, Gao, et al., 2013). How cancer alters the fasting regulation of muscle AMPK signaling remains to be determined. While decreased physical activity occurs before cachexia development (Baltgalvis et al., 2010), it is unknown if increased physical activity during cachexia initiation can improve muscle AMPK’s response to fasting. Overall, there is an incomplete understanding of muscle AMPK’s role during cachexia initiation and if exercise could improve this response. Therefore, we examined if voluntary wheel exercise could improve skeletal muscle AMPK signaling in pre‐cachectic MIN mice. Next, we examined muscle AMPK’s role in aberrant catabolic signaling in response to a 12‐h fast in MIN mice initiating cachexia. Male MIN mice initiating cachexia were assigned to either *ad libitum* feeding, a 12‐hour fast, or a 12‐hour fast after 4 weeks of running wheel access. Next, we examined AMPK’s regulation of fasting in MIN mice. Male tamoxifen‐inducible skeletal muscle AMPKα ***^1^*** α ***^2^*** (KO) knockout mice crossed with *Apc^Min^* ^/+^ mice and floxed controls were examined during cachexia progression and after a 12‐hour fast.

### 1.1 Methods

Male C57BL/6 and *Apc^Min^* ^/+^ (MIN) mice were purchased from Jackson Laboratories and were bred at the University of South Carolina's Animal Resources Facility. All animals were group‐housed and kept on a 12:12‐h light–dark cycle. Body weights were measured weekly, and animals were monitored for signs of distress. Animals were given food and water ad libitum until sacrifice.

### 1.2 Experiment 1: Effect of wheel running on fasting‐induced skeletal muscle AMPK signaling

Male C57BL/6 (wild type; B6) and *Apc^Min^* ^/+^ (MIN) were randomly assigned to one of the three conditions and at the end of the study were sacrificed either ad libitum, fast, or wheel +fast. To allow for a fed state, mice in the ad libitum condition were sacrificed at the end of the dark cycle in the ad libitum condition. To examine the fasted state, mice were fasted at the start of the light cycle and sacrificed at the beginning of the dark cycle allowing for a 12‐hour fast. A 12‐h fast is common method to standardize testing outcomes to reduce variability (Jensen et al., 2013) and different from a starvation model which utilizes 24‐, 48‐, or 72‐h fast (Bujak et al., 2015; Castets et al., 2013). To examine the role of wheel running (wheel + fast), mice were allowed free access to a wheel for 4 weeks, and mice were fasted at the start of the light cycle and sacrificed at the beginning of the dark cycle allowing for a 12‐h fast. B6 mice between 12 and 19 weeks of age were used for these experiments.

### 1.3 Voluntary wheel running—experiment 1

Voluntary wheel running was used as volitional physical activity and performed as previously described (Baltgalvis et al., 2010). At ~14 to 15 weeks of age, B6 and MIN mice were housed individually in cages with 9.5‐in. diameter stainless steel activity wheels (MiniMitter, Bend, OR). Running activity was monitored daily, starting at 14–15 weeks of age to 18–19 weeks of age. Bicycle computers (Specialized, Morgan Hill, CA) with magnetic sensors measured average speed, distance, time, and maximum speed, and the data were recorded daily. Wheels were removed from the mouse's cage 72 h before sacrifice.

### 1.4 *Experiment 2*: AMPK’S regulation of fasting in tumor‐bearing mice

To examine the regulation of AMPK loss in tumor‐bearing mice, AMPKα ***^1^*** α ***^2^*** knockout mice and floxed controls were generated *(see next section for details)*. Between 15 and 17 weeks of age, male AMPKα ***^1^*** α ***^2^*** knockout or floxed controls in the WT and MIN genotype were fasted at the start of the light cycle and sacrificed at the beginning of the dark cycle allowing for a 12‐h fast.

### 1.5 Generation of AMPKα^1^α^2^ skeletal muscle deletion

To generate a skeletal muscle‐specific knockout of AMPKα ***^1^*** α ***^2^***, mice that contained individually floxed alleles for AMPKα ***^1^*** and AMPKα ***^2^*** were a kind gift from Dr. Hoh‐Jin Koh at the University of South Carolina. The AMPKα ***^1^*** α ***^2^*** was then crossed with *Apc^Min^* ^/+^ mice to generate an AMPKα ***^1^*** α ***^2^*** *Apc^Min^* ^/+^ mouse. Utilizing the skeletal actin (HSA) promoter‐driven expression of a Cre recombinase flanked by mutated estrogen receptors (HSA‐MCM) (McCarthy et al., 2012). Tamoxifen‐inducible Mer Cre Mer driven by a human skeletal actin promoter (HSA) mouse was purchased from Jackson Laboratories (Bar Harbor). Male tamoxifen‐inducible skeletal muscle AMPKα ***^1^*** α ***^2^*** (KO) knockout mice and floxed (Flox) controls were produced. This genetic approach provides viable, healthy mice, and has been successfully used by others to study muscle‐specific inducible loss of AMPK in skeletal muscle (Hingst et al., 2020; Lantier et al., 2020). Finally, the AMPKα(Argiles et al., 2010) α ***^2^*** HSA Cre mice were crossed with AMPKα ***^1^*** α ***^2^*** *Apc^Min^* ^/+^ mice giving a 50% chance of the offspring producing an AMPKα ***^1^*** α ***^2^*** *Apc^Min^* ^/+^ HSA Cre mouse. At approximately 12 weeks of age, all AMPK KO and floxed control mice received tamoxifen injection (i.p. 2 mg) daily for five consecutive days (Harfmann et al., 2016), and then underwent a 2‐week washout period before beginning the experiment (Figure 5).

### 1.6 Tissue collection

Mice were euthanized with a subcutaneous injection of a ketamine‐xylazine‐acepromazine cocktail (1.4 mg/kg body weight) (Baltgalvis et al., 2010). Muscles and organs were rapidly excised, cleared for excessive connective tissue, weighed, and snap‐frozen in liquid nitrogen.

## 2 INTESTINAL POLYP QUANTIFICATION

Intestinal segments were excised, cleaned with PBS, cut into equal segments, and stored in 10% neutral formalin until tumor count analysis. Intestinal polyps were analyzed after a deionized water rinse and 0.1% methylene blue staining. Total polyp counts were performed using dissecting micro‐scope (model SMZ168, Motic, Xiamen, China) by an investigator blinded to the treatment.

### 2.1 Western blotting

Western blot analysis was performed as previously described (Hardee, Fix, et al., 2018). Briefly, frozen gastrocnemius muscle was homogenized in ice‐cold Mueller buffer, and protein concentration was determined by the Bradford method. Crude muscle homogenates were fractionated on 8–15% SDS‐polyacrylamide gels and transferred to PVDF membranes overnight. Membranes were stained with Ponceau red to verify equal loading and transfer for each gel. Membranes were blocked at room temperature for 1h in 5% Tris‐buffered saline with 0.1% Tween‐20 (TBST) milk. Primary antibodies for p‐FOXO3a (S413, #8174), FOXO3a (#12829), p‐ULK‐1 (S555,#5869) (S757,#14202), ULK‐1 (#8054), P62 (#23214), LC3B (#3868), p‐AMPK (T172, #50081), AMPK (#2532), p‐ACC (S79, #1181), ACC (#3676), DRP‐1 (#8570), and MFN‐1 (#14739) were purchased from cell signaling and diluted 1:500–1:2000 in 5% TBST‐milk followed by overnight incubation with membranes at 4 degrees. Primary antibodies for MuRF‐1 (ECM Biosciences, #MP3401), Atrogin‐1 (ECM Biosciences, #AP2041), and PGC‐1α (Abcam, #ab54481) were diluted 1:1000–1:2000 in 5% TBST‐milk followed by overnight incubation with membranes at 4 degrees. Anti‐rabbit or anti‐mouse IgG horseradish‐peroxidase conjugated secondary antibody (Cell Signaling) was incubated with the membranes at 1:4000 dilution for 1 hour in 5% TBST‐milk at room temperature. Enhanced chemiluminescence (ECL) (GE Healthcare Life Sciences, Piscataway, NJ) was used to visualize the antibody‐antigen interactions. Images were digitally scanned and quantified by densitometry using imaging software (Image J; NIH).

### 2.2 Cytochrome C oxidase enzyme assay

Cytochrome‐c oxidase (COX) activity was assessed in whole muscle gastrocnemius homogenates. Gastrocnemius muscle was homogenized in extraction buffer (0.1 M KH2PO4/Na2HPO4 and 2 mM EDTA, pH 7.2). COX enzyme activity was determined by measuring the rate of oxidation of fully reduced cytochrome c at 550 nm as previously described (Fix et al., 2018).

### 2.3 Plasma IL‐6 concentrations

Plasma interleukin‐6 (IL‐6) concentrations were determined as previously described (Hardee, Mangum, et al., (1985). Immediately prior to sacrifice, blood was collected via retro‐orbital sinus with heparinized capillary tubes, placed on ice, and centrifuged (10,000 × g for 10 min at 4℃). The supernatant was removed, and plasma IL‐6 concentration was determined. A commercially available IL‐6 enzyme‐linked immunosorbent assay kit (BD Biosciences) was used and the manufacturer's protocol was followed. Briefly, a Costar transparent 96‐well plate (Corning, NY, USA) was coated with IL‐6 capture antibody and allowed to incubate overnight. The next morning, the plate was blocked with assay diluent buffer, washed, and IL‐6 standards, and plasma samples were added in duplicate to the plate. The plate was again washed, and the sAV‐HRP reagent was added to each well. After several washes, the TMB substrate was added, and the reaction was developed for 20 min. The reaction was stopped with sulfuric acid, and absorbance was read in a Bio‐Rad iMark plate reader (Hercules) at 450 nm.

### 2.4 Statistical analysis

Results are reported as the means ± *SE*. In experiment 1, we used two‐way ANOVA (2 genotypes × 3 treatments) to compare B6 and MIN mice in the *ad libitum*, fast, and wheel + fast conditions. We used an unpaired Student's preplanned *t*‐test to compare daily wheel distance in B6 and MIN mice given wheel access. Pearson Correlation was used to compare COX enzyme activity to distance per day in MIN Wheel + Fast Mice. Post‐hoc analyses were performed with Tukey's test when appropriate. In experiment 2, we used a two‐way ANOVA to compare WT and MIN mice in the AMPKα ***^1^*** α ***^2^*** knockout and floxed controls. Post hoc analyses were performed with Tukey's test when appropriate. The accepted level of significance was set at *p* < 0.05 for all analyses. All statistical analyses used Prism GraphPad 7 (GraphPad Software Inc.).

## 3 RESULTS

### 3.1 Mouse characteristics and wheel running activity—Experiment 1

**FIGURE 1:**
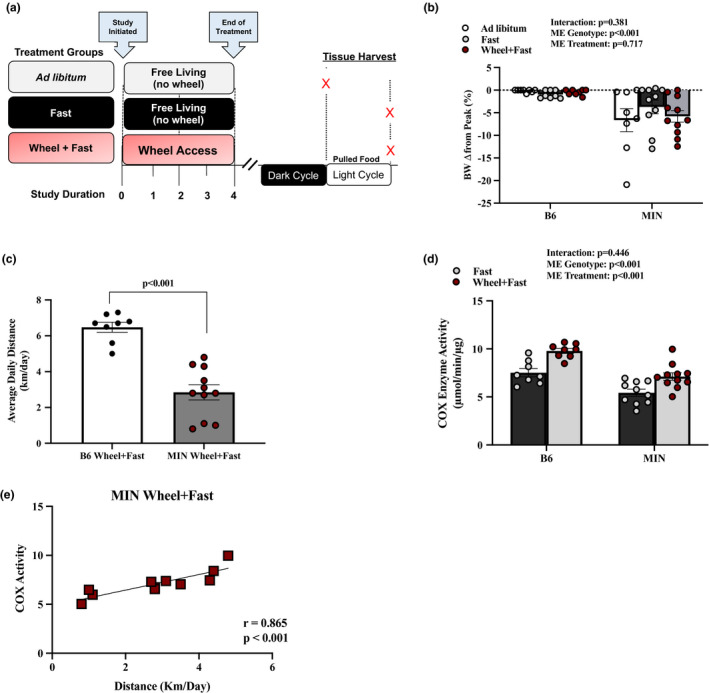
Effect of Fasting and Wheel Running on Animal Characteristics, Activity, and COX Activity in MIN Mice—Experiment 1. Data are expressed as mean ± SEM. a) Study design. b) Body weight change from peak. c) Average daily wheel distance in Wheel +Fast group. d) COX Enzyme Activity in Fast and Wheel +Fast B6 and MIN Mice. e) COX Enzyme activity association to distance run in MIN Wheel +Fast Mice. Abbreviations: mg/dl: milligrams per deciliters, km/day: kilometers per day, µmol/min/µg: micromole per minute per ug of tissue. Two‐way ANOVA was used to compare B6 and MIN mice in the three treatment groups (b) and COX enzyme activity (d). Unpaired *t*‐test was used to compare average daily wheel distance (c). Pearson Correlation was used to compare COX enzyme activity to distance per day in MIN Wheel +Fast Mice (e). Statistical significance is *p* < 0.05. *N* = 8–11 per group

Muscle signaling following wheel‐running was examined in B6 and MIN mice randomly assigned to either ad libitum (no wheel access), a 12‐h fast (no wheel access), or free access to a wheel for 4 weeks and then subjected to a 12‐h fast (Figure 1a). There were no significant differences in peak BW, pre‐sacrifice BW, or hindlimb muscle mass in the three B6 treatment groups (Table 1). MIN mice had elevated plasma IL‐6, increased spleen mass, reduced gastrocnemius muscle mass, decreased seminal vesicle, and epididymal fat mass (Table 1) compared to B6. While MIN mice exhibited body weight loss compared to B6 mice, MIN mice had moderate body weight loss characterizing them as pre‐cachectic tumor bearing mice (Figure 1b). There were no differences in total polyp number between MIN treatment groups.

**TABLE 1:** Effect of Fasting and Wheel Running on Animal Characteristics in B6 and MIN mice

	B6			MIN			
Treatment	Ad libitum	Fast	Wheel+Fast	*Ad libitum*	Fast	Wheel+Fast	ME
N	8	8	10	8	10	11	
BW pre‐sacrifice (g)	25.1 (0.3)	23.6 (0.5)	24.6 (0.4)	21.8 (0.4)^^^	23.1 (0.5)^^^	23.2 (0.3)^$^	
Peak BW (g)	25.1 (0.3)	23.9 (0.5)	24.7 (0.4)	23.2 (0.8)	24.0 (0.4)	24.5 (0.3)	
*Cachexia Indices*							
Plasma IL−6 (pg/ml)	N.D	N.D	N.D	45 (7)	23 (4)	25.6 (18)	^@^
Polyp count (#)	‐	‐	‐	59 (4)	40 (7)	49 (4)	
Spleen (mg)	159 (23)	142 (15)	69 (4)	387 (35)	344 (39)	310 (33)	^+@^
Gastrocnemius mass (mg)	123 (2)	122 (3)	127 (2)	89 (6)	100 (8)	117 (5)	^+@^
Seminal ves. mass (mg)	190 (13)	209 (9)	249 (11)	85 (18)	131 (25)	140 (14)	^+@^
eWAT mass (mg)	277 (21)	290 (26)	252 (11)	49 (25)	99 (39)	10 (23)	^@^
Liver mass (mg)	1495 (45)	1043 (14)	1136 (31)	1454 (149)	1287 (57)	1421 (70)	^+@^
Stomach mass (mg)	558 (44)	259 (23)	178 (19)	556 (69)	424 (38)	392 (58)	^+@^
Blood glucose @ sacrifice (mg/dl)	122 (5)	128 (4)	132 (3)	120 (5)	113 (5)	120 (6)	^@^
Food intake (avg. g/day)	3.6 (0.2)	3.3 (0.3)	4.2 (0.1)	2.1 (0.3)	2.9 (0.3)	3.9 (0.2)	^+@^
Tibia length (mm)	16.8 (0.1)	16.7 (0.1)	16.7 (0.1)	16.9 (0.1)	16.8 (0.1)	16.6 (0.1)	

Data are expressed as mean (SEM). Animal characteristics were measured at the end of the study.

Abbreviations: #, number; %, percent; g/day, grams per day; g, grams; mg, milligrams; mm, millimeters; N.A., Not applicable; pg/ml, picograms per milliliter; wks., weeks.

Food intake is the average food intake over a three‐day period prior to sacrifice. Main Effects (ME): @ME Genotype and +ME Treatment. Interaction Significance: # Different from all groups, ^^^ Different from B6 ad libitum, ^&^ Different from MIN ad libitum, $ Different from B6 Wheel+Fast.

Overall wheel running increased food intake, reduced spleen mass, increased seminal vesicle mass in both B6 and MIN mice compared to *ad libitum* and fast treatment groups (Table 1). Wheel running increased total hindlimb muscle mass in both B6 and MIN mice compared to the ad libitum groups. MIN mice with wheel access ran significantly less than B6 mice with wheel access (Figure 1c). While there was a main effect for MIN mice to have decreased muscle COX activity compared to B6 mice, there was also a main effect for wheel access to increase muscle COX activity (Figure 1d). MIN muscle COX activity was significantly associated with wheel distance run (km/day) during the study (Figure 1e).

### 3.2 Effect of a fast on muscle AMPK SIGNALING in MIN Mice—Experiment 1

**FIGURE 2:**
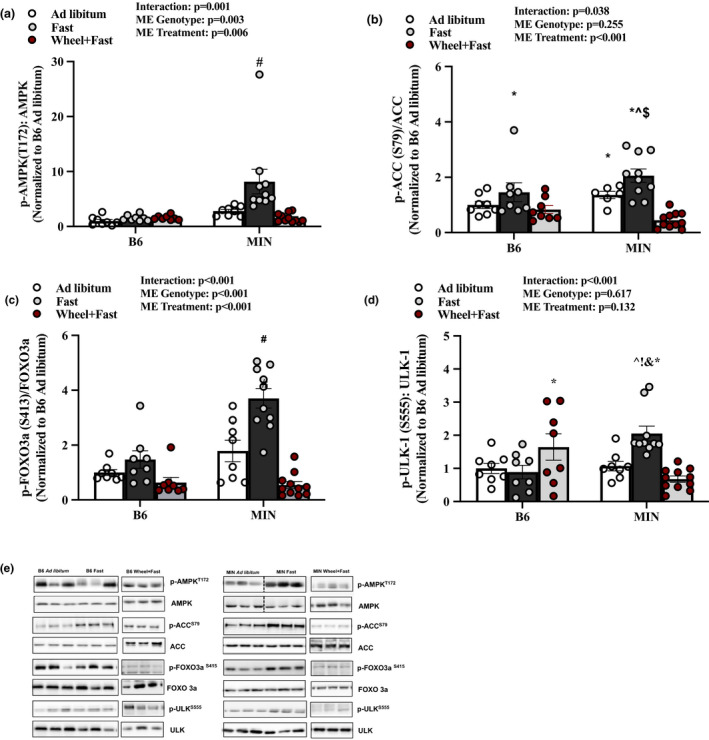
Effect of a Fast on Muscle AMPK Signaling in MIN Mice—Experiment 1. Data are expressed as mean ± SEM. a) pAMPK T172/AMPK; b) pACC S79/ACC; c) pFOXO S413/FOXO3a; d) pULK‐1S555/ULK; e) Representative western Blots. Gastrocnemius muscle protein expression in B6 and MIN Mice sacrificed in the ad libitum, fast, or Wheel +Fast condition normalized to B6 ad libitum. Cropped lines indicate that the samples were run on the same gel and solid lines means samples were transposed across gels. Abbreviations: AMPK: AMP‐activated protein kinase, ACC: acetyl CoA carboxylase, FOXO: Forkhead Box O, ULK: Unc‐51‐like kinase 1. Two‐way ANOVA was used to compare B6 and MIN mice in the three treatment groups. Interaction Symbols: # Different from all groups; ^ different from B6 ad libitum; & different from MIN ad libitum;! different from B6 fast; $ different from B6 wheel+fast; * different from MIN wheel+fast. Statistical significance is *p* < 0.05. *N* = 8–11 per group

In B6 mice, AMPK phosphorylation (T172) and downstream target ACC (S79) were not different between the three B6 treatment groups (Figure 2a). MIN mice subjected to a 12‐h fast demonstrated a significant induction of muscle AMPK phosphorylation (T172) compared to all groups (Figure 2a). MIN mice subjected to a 12‐h fast increased AMPK downstream target ACC (S79) compared to B6 ad libitum and B6 wheel + fast (Figure 2b). Wheel running suppressed the fasting induction of muscle AMPK and ACC phosphorylation in MIN mice (Figure 2a,b). Downstream AMPK phosphorylation target FOXO3a (S413) was significantly increased in MIN fast compared to all groups and wheel running significantly reduced FOXO3a (S413) compared to MIN ad libitum and fast (Figure 2c). ULK‐1 (S555), a downstream target of AMPK, was not altered in the B6 by the three treatment groups. Fasting significantly increased MIN ULK‐1 (S555) compared to B6 fast and MIN ad libitum, and wheel running was sufficient to lower this response (Figure 2d). These results provide evidence that MIN mouse muscle AMPK signaling has greater sensitivity to a 12 h fast than B6 mice, and wheel running is sufficient to lower this fasting response.

### 3.3 Effect of a fast on skeletal muscle E3 ligase and autophagy‐associated proteins in MIN mice—Experiment 1

**FIGURE 3:**
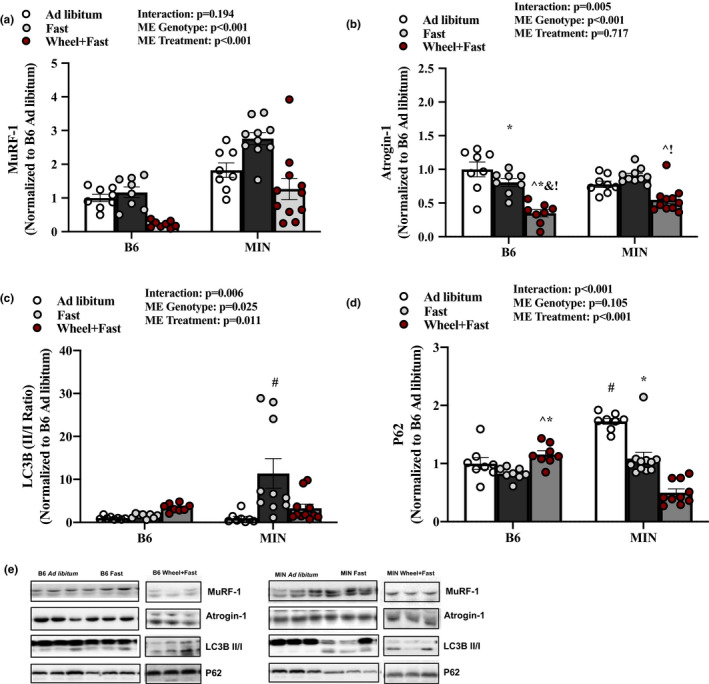
Effect of a Fast on Skeletal Muscle E3 Ligase and Autophagy‐Associated Proteins in MIN mice—Experiment 1. Data are expressed as mean ±SEM. a) MuRF‐1; b) Atrogin‐1; c) LC3B II/I ratio; d) P62; e) Representative western Blots. Gastrocnemius muscle protein expression in B6 and MIN Mice sacrificed in the ad libitum, fast, or Wheel +Fast condition normalized to B6 ad libitum. Cropped lines indicate that the samples were run on the same gel and solid lines means samples were transposed across gels. Abbreviations: LC3B: microtubule‐associated proteins 1A/1B light chain 3B. Two‐way ANOVA was used to compare B6 and MIN mice in the three treatment groups. Interaction Symbols: # different from all groups; ^ different from B6 ad libitum; & different from MIN ad libitum;! different from B6 fast; $ different from B6 wheel+fast; * different from MIN wheel+fast. Statistical significance is *p* < 0.05. *N* = 8–11 per group

AMPK is an upstream regulator of skeletal muscle E3 ligases and lysosomal autophagy. We examined if wheel running could improve the fasting regulation of these proteins. Overall, MIN mice had increased MuRF‐1 protein expression (Figure 3a). A 12‐h fast increased MuRF‐1 protein expression and wheel running was sufficient to lower this response (Figure 3a). A 12‐h fast and MIN mice did not induce Atrogin‐1 protein expression (Figure 3b). In B6 mice, wheel running significantly suppressed muscle Atrogin‐1 expression compared B6 fast and ad libitum (Figure 3b). LC3B and P62 are autophagy‐associated proteins. In B6 mice, fasting did not alter LC3B II/I ratio or P62 protein expression. In the MIN, fasting produced a ninefold induction of muscle LC3B II/I ratio (Figure 3c). MIN in the ad libitum condition induced P62 compared to B6 fast (Figure 3d). While a 12‐h fast did not alter MIN muscle P62 protein expression, wheel running did suppress P62 expression (Figure 3d). Collectively these data suggest that in the MIN mouse, E3 ligase and autophagy‐associated proteins are sensitive to a fast, and wheel running activity can attenuate this sensitivity to fasting.

### 3.4 Effect of a fast on mitochondrial quality control proteins in MIN mice—Experiment 1

**FIGURE 4:**
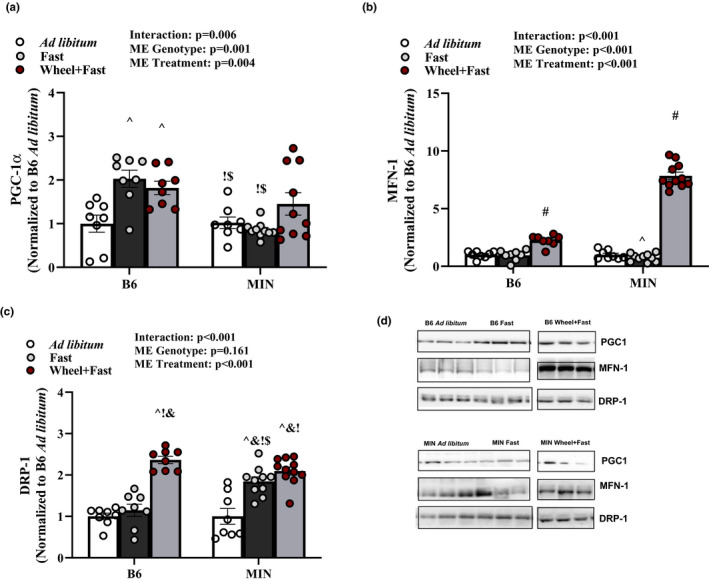
Effect of a Fast on Mitochondrial Quality Control Proteins in MIN Mice—Experiment 1. Data are expressed as mean ± SEM. a) PGC‐1 α; b) MFN‐1; c) DRP‐1; d) Representative western blots. Gastrocnemius muscle protein expression in B6 and MIN Mice sacrificed in the ad libitum, fast, or Wheel +Fast condition normalized to B6 ad libitum. Cropped lines indicate that the samples were run on the same gel and solid lines means samples were transposed across gels. Abbreviations: PGC‐1 α: peroxisome‐proliferator gamma‐activated receptor coactivator, MFN‐1: mitofusion, and DRP: dynamin‐related protein. Two‐way ANOVA was used to compare B6 and MIN mice in the three treatment groups. Interaction Symbols: # different from all groups; ^ different from B6 ad libitum; & different from MIN ad libitum;! different from B6 fast; $ different from B6 wheel+fast; *different from MIN wheel+fast. Statistical significance is *p* < 0.05. *N* = 8–11 per group

AMPK has emerged as a critical regulator of muscle mitochondrial health by its regulation of mitochondrial biogenesis (PGC‐1α) and fission (DRP‐1) (Chen et al., 2019). These processes are essential for skeletal muscle maintenance during periods of fasting and acute exercise. Therefore, we examined the effect of a 12‐h fast and voluntary wheel running on mitochondrial quality control proteins. In B6 mice, muscle PGC‐1α expression was increased compared to ad libitum B6 mice. In the MIN ad libitum and fast, muscle PGC‐1α expression was decreased compared to B6 fast and wheel+fast, without differences between MIN treatment groups (Figure 4a). MFN‐1 was significantly increased in both B6 and MIN mice given wheel access compared to all other groups (Figure 4b). DRP‐1 were both significantly increased in B6 mice given wheel access compared to B6 ad libitum and fast (Figure 4c). Fasting increased MIN muscle DRP‐1 expression compared to B6 ad libitum and fast and MIN ad libitum, and wheel running in the MIN did not alter this response (Figure 4c). These results collectively suggest that wheel running increases MIN muscle mitochondrial fission and fusion proteins DRP‐1 and MFN‐1 expression.

### 3.5 Effect of AMPK loss on muscle signaling in MIN mice—Experiment 2

**FIGURE 5:**
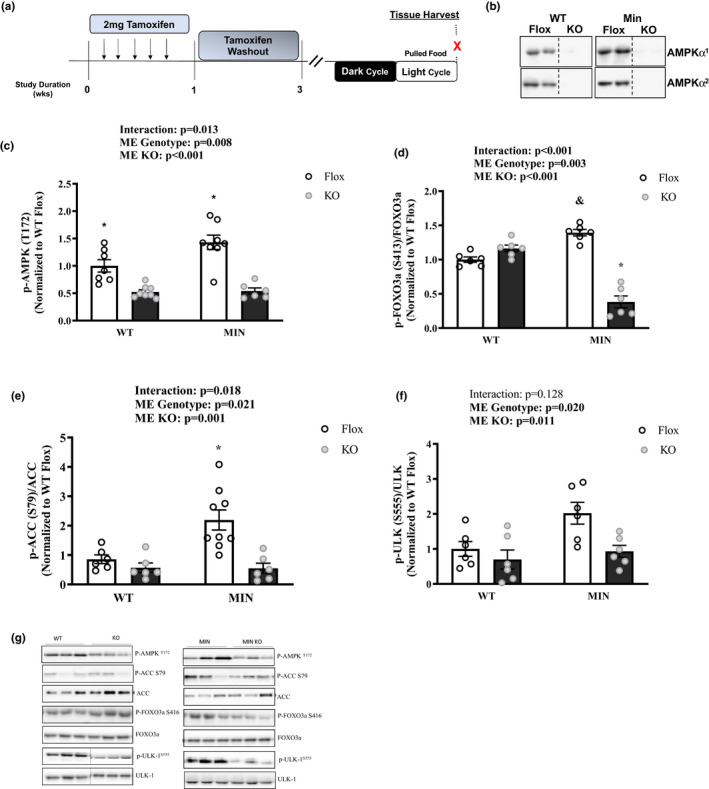
Effect of AMPK Loss on Muscle Signaling in MIN Mice—Experiment 2. Data are expressed as Mean ±SEM. a) Study Design. All mice were sacrificed at the end of the light cycle following a 12‐hr fast. b) Total AMPK α*^1^* and AMPK α*^2^* in C57Bl/6 (WT) and *Apc* *^Min^* ^/+^ (MIN) mice. Cropped lines indicates that the samples were run on the same gel, just not right beside each other. c) pAMPK T172/AMPK; d) pFOXO S413/FOXO3a; e) pACC S79/ACC; f) pULK‐1S555/ULK; g) Representative Western Blots. Gastrocnemius protein expression. Cropped lines indicate that the samples were run on the same gel and solid lines means samples were transposed across gels. Abbreviations: AMPK: AMP‐activated protein kinase, ACC: acetyl CoA carboxylase, FOXO: Forkhead Box O, ULK: Unc‐51‐like kinase 1. Two‐way ANOVA was used to compare between WT and MIN AMPKα*^1^* α*^2^* floxed or muscle specific KO mice in the fasted condition. Statistical significance is *p* < 0.05. *Different from all groups; & different from WT Flox. *N* = 6–10 per group

**FIGURE 6:**
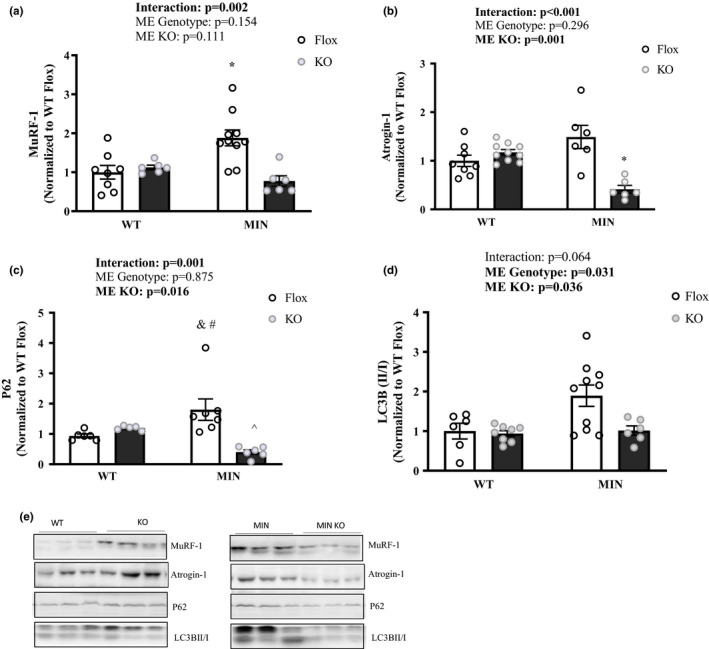
The Effect of AMPK Loss on Fasting's Regulation of Skeletal Muscle Signaling– Experiment 2. Data are expressed as Mean ± SEM. All mice were sacrificed at the end of the light cycle following a 12‐h fast. a) MuRF‐1; b) Atrogin‐1; c) P62; d) LC3B II/I ratio; e) Representative western Blots. Gastrocnemius protein expression. Cropped lines indicate that the samples were run on the same gel and solid lines means samples were transposed across gels. Abbreviations: LC3B: Microtubule‐associated proteins 1A/1B light chain. Two‐way ANOVA was used to compare between WT and MIN AMPKα*^1^* α*^2^* floxed or muscle specific KO mice in the fasted condition. Statistical significance is *p* < 0.05. * Different from all groups; ^ different from WT KO; & different from WT Flox; # different than MIN KO. *N* = 6–10 per group

To determine AMPK’s role in muscle's response to a 12‐h fast during cachexia, we examined inducible skeletal muscle AMPKα^1^ and AMPKα^2^ loss in MIN mice (MIN KO). Daily tamoxifen injection for five consecutive days followed by a 2‐week washout period was sufficient to induce human skeletal actin (HSA) cre deletion of AMPKα^1^ and AMPKα^2^ from skeletal muscle in both WT and MIN mice (Figure 5a,b). AMPK KO did not prevent body weight loss or muscle mass loss (Table 2). As expected, the MIN KO mice had suppressed AMPK (T172), FOXO3a (S413), and ACC (S79) (Figure 5c–e). There was a main effect for MIN mice to have increased ULK1 (S555) activation, and a main effect for AMPK loss to decrease ULK1 (S555) activation (Figure 5f). We examined if MIN KO mice had altered regulation of skeletal catabolism signaling. The MIN KO mice had a suppressed fasting induction of MuRF‐1 and Atrogin‐1 protein expression (Figure 6a,b). P62 protein expression was also suppressed in MIN KO mice (Figure 6c). There was a main effect for MIN mice to have increased LC3B II/I expression, and a main effect for AMPK loss to decease LC3B II/I expression (Figure 6d). Together these data demonstrate that AMPK activity is at least partially responsible for the 12h fasting induction of catabolic signaling in MIN mice.

**TABLE 2:** Effect of muscle specific AMPK loss on animal characteristics in MIN mice

	WT	KO		MIN	MIN KO
N	8	8		10	6
Age @ sacrifice (wks.)	16.1 (0.2)	15.9 (0.1)		21.3 (1.0)*	19.0 (1.3)*
Peak BW (g)	23.5 (0.5)	23.2 (0.5)		24.1 (0.4)	23.8 (0.7)
BW pre‐sacrifice (g)	23.5 (0.5)	22.7 (0.6)		22.9 (0.8)	22.7 (0.6)
BWΔ from peak (%)	−0.1 (0.0)	−2.2 (0.5)		−5.1 (2.3)*	−4.6 (1.2)*
Sac glucose (mg/dL)	141 (7)	126 (7)		113 (5)^	133 (11)
Food (g/day)	3.4 (0.2)	3.2 ± 0.1		2.9 (0.3)	3.1 (0.2)
IL−6 (pg/mL)	N.D.	N.D.		23 (4)*	18 (4)*
Total polyps (#)	N.D.	N.D.		40 (7)*	37 (10)*
Gastrocnemius (mg)	120 (3)	119 (2)		100 (7)*	120 (4)*
Seminal vesicles (mg)	171 (12)	155 (14)		131 (24)	166 (19)
eWAT (mg)	237 (30)	237 (22)		99 (39)*	202 (15)*
Stomach mass (mg)	251 (22)	258 (10)		424 (38)*	283 (22)*
Liver mass (mg)	1007 (41)	971 (27)		1287 (57)*	1040 (105)*
Spleen mass (mg)	61 (2)	84 (17)		344 (39)*	151 (30)*
Tibia length (mm)	16.9 (0.1)	17.1 (0.1)		16.8 (0.1)	16.9 (0.1)

Data are expressed as Mean (SEM). Animal characteristics were measured at the end of the study.

Two‐way ANOVA was used to compare between WT and MIN and AMPK KO. Statistical significance is *p* < 0.05.

Abbreviations: #, number; %, percent; eWAT, epididymal fat mass; g/day, grams per day; g, grams; mg, milligrams; mm, millimeters; ND, not detected; pg/ml, picograms per milliliter; wks., weeks.

* ME (Main Effect) of MIN.

### 3.6 Effect of AMPK loss on muscle mitochondrial quality control proteins in MIN mice—Experiment 2

**FIGURE 7:**
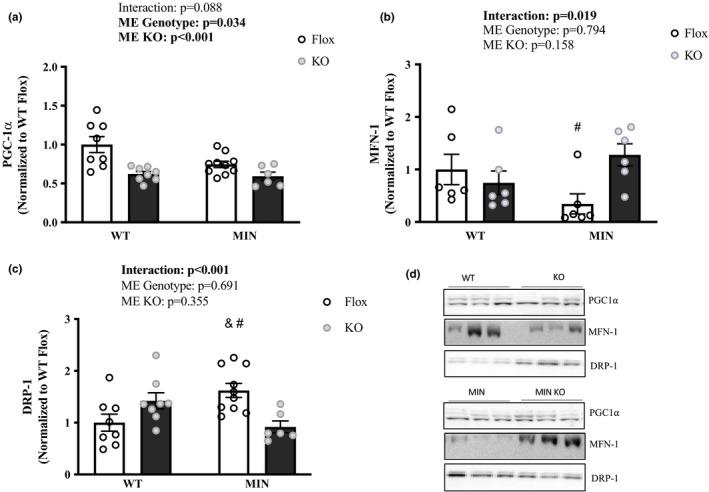
Effect of AMPK Loss on Muscle Mitochondrial Quality Control Proteins in MIN Mice—Experiment 2. Data are expressed as mean ± SEM. All mice were sacrificed at the end of the light cycle following a 12‐h fast. a) PGC‐1 α; b) MFN‐1; c) DRP‐1; d) Representative western Blots. Gastrocnemius protein expression. Cropped lines indicate that the samples were run on the same gel and solid lines means samples were transposed across gels. Abbreviations: PGC‐1 α: peroxisome‐proliferator gamma‐activated receptor coactivator, MFN‐1: mitofusion, and DRP: dynamin‐related protein. Two‐way ANOVA was used to compare between WT and MIN AMPKα*^1^* α*^2^* floxed or muscle specific KO mice in the fasted condition. Statistical significance is *p* < 0.05. *Different from all groups; & different from WT Flox; # different than MIN KO; ^ different from WT KO. *N* = 6–10 per group

Lastly, we determined if skeletal muscle loss of AMPK in MIN mice regulated mitochondrial quality control proteins. There was a main effect for MIN mice to have lower PGC‐1α protein expression and AMPK loss to further lower PGC‐1α (Figure 7a). Surprisingly, AMPK loss restored MFN‐1 protein expression and attenuated the induction of DRP‐1 (Figure 7b,c). These results suggest that AMPK is necessary to suppress MFN‐1 and induce DRP‐1 in 12‐h fasted MIN skeletal muscle.

### 3.7 Discussion

Muscle AMPK signaling has an established role in regulating metabolism and muscle protein turnover in response to metabolic challenges. Specifically, an acute bout of exercise induces short‐term activation of AMPK that can initiate glucose uptake, fatty acid oxidation, and mitochondrial biogenesis (Egan & Zierath, 2013). Regular exercise can prevent the chronic induction of muscle AMPK in tumor‐bearing mice (Puppa et al., 2012), further underscoring exercise's effect in preventing indices of cachexia (Coletti et al., 2016; Mehl et al., 2005; Pigna et al., 2016; Vanderveen et al., 2020; Jee et al., 2016). Therefore, aberrant AMPK activity could be involved in muscle mass loss with cancer cachexia (Kemp et al., 2007; Koh et al., 2008b). We have previously reported that skeletal muscle AMPK is chronically activated in cachectic mice (Hardee, Fix, et al., 2018; Hardee, Mangum, et al., (1985); White, Puppa, Gao, et al., 2013). We now report the novel finding that muscle AMPK dysregulation to a 12‐h fast is an early event in cachexia progression. We also report that voluntary wheel running, prior to cachexia development, can suppress the fasting‐induced activation of AMPK signaling. Moreover, our results indicate that the fasting induction of FOXO3a, ACC, and MuRF‐1 requires AMPK, as does the fasting regulation of mitochondrial quality control proteins MFN‐1 and DRP‐1 in tumor‐bearing mice.

AMPK is critical for maintaining energy status and metabolic homeostasis. In times of energy deficiency or stress (fasting), AMPK will activate energy‐generating pathways such as the FOXO3a controlled E3 ligases, or the ULK‐1 mediated autophagy–lysosomal system (Bujak et al., 2015; Mihaylova & Shaw, 2011). The phosphorylation of TSC2 and Raptor by AMPK can suppress the metabolically demanding protein synthesis process (Corradetti et al., 2004; Gwinn et al., 2008; Shaw, 2009). The suppression of mTORC1 activity and muscle protein synthesis may be related to the chronic induction of muscle AMPK that occurs with cachexia (White, Puppa, Gao, et al., 2013). Although, many of these earlier studies failed to consider the nutrient status and activity on the AMPK signaling pathway in cachectic skeletal muscle. Phosphorylation of ULK‐1 at S555 by AMPK is critical in initiating the autophagy process (Bujak et al., 2015; Mihaylova & Shaw, 2011; Sanchez et al., 2012a; Zhao & Klionsky, 2011). AMPK and mTORC1 both regulate ULK‐1 and have competing effects through phosphorylation events (Kim et al., 2011). Unfortunately, there are only a few studies that have examined the impact of cancer cachexia progression on AMPK regulation in skeletal muscle. AMPK activators, such as AICAR and Metformin administered before cachexia initiation, have been examined with some preclinical models of cancer cachexia. AICAR administration prevented muscle mass loss in tumor‐bearing mice (Hall et al., 2018; Pigna et al., 2016) and lowered E3 ligase gene expression (Pigna et al., 2016). Additionally, metformin also prevented indices of cachexia (Hall et al., 2018; Oliveira & Gomes‐Marcondes, 2016). Our study is in contrast to many because we chose to reduce AMPK while there are several known benefits of inducing AMPK. It is necessary to put into context the role of AMPK, we chose to lower the already elevated AMPK in cachectic skeletal muscle to examine its regulation during a 12‐h fast. Previous publications have used non‐tissue specific activators of AMPK administered prior to or at the start of tumor inoculation. These studies provide great insight into the understanding of AMPK activation at the initiation of tumor development and its implications on the later development of cachexia. Furthermore, cancer cachexia is a multiorgan condition; therefore, it is possible that AMPK activation in tissues other than muscle benefit from increased AMPK signaling at the onset of tumor development. It is interesting to speculate the effects of these AMPK activators once cachexia has developed. Alternatively, we chose to lower AMPK once the mouse had the tumor environment for several weeks and was known to have elevated muscle AMPK. To date, we have only started to understand the effects of AMPK on cancer cachexia and future studies should delineate the importance of AMPK early compared to late‐stage cachexia.

The IL‐6/gp130 signaling pathway in preclinical models and human cancer patients has linked autophagy regulation to cancer‐induced inflammation (Aversa et al., 2016; Penna et al., 2013; Pettersen et al., 2017). Recent studies in C26 tumor‐bearing mice demonstrated the AMPK dependence of elevated autophagy (Penna et al., 2013). Severely cachectic MIN mice have increased muscle Beclin‐1 and LC3B expression (White et al., 2012), suggesting that more severe cachexia is associated with higher autophagy–lysosome system activity (White et al., 2011). In the current study, we demonstrate that the induction of autophagy‐related signaling following a 12‐h fast is suppressed by wheel running is an early event in cachexia's progression. Interestingly, we observe an increase in P62 without an associated increase in LC3BII/I in the MIN ad libitum, suggestive of autophagy inhibition. While we did sacrifice ad libitum mice at the end of the dark cycle to utilize the fed condition, it is possible that the circadian rhythms can play a role in the intracellular signaling and should be considered. Furthermore, it is interesting to hypothesize that the cancer environment disrupts the feeding and fasting regulation of autophagy which can be rescued by physical activity; however future studies are needed in order to clarify if and where in the autophagy process these disrupts might be occurring (Buch et al., 2020; Yoshii & Mizushima, 2017). We build on these findings by reporting that the fasting induction autophagy‐related signaling in MIN mice requires AMPK and works through ULK‐1 phosphorylation. Both wheel running and loss of muscle AMPK in MIN mice suppressed ULK‐1 and downstream target P62. It is important to mention that wheel running increased muscle mass in tumor bearing mice which has been previously report (Pigna et al., 2016; Vanderveen et al., 2020). Additionally, AMPK loss did not affect muscle mass weight. Previous publications have reported the loss of AMPK either whole body or heart and muscle does decrease muscle cross sectional area (Kjobsted et al., 2018; Thomas et al., 2014). Additionally, it is important to consider the duration of AMPK loss. Herein we examined the mechanistic role of AMPK loss after 2 weeks, whereas other studies examined the role of AMPK loss after 4–8 weeks. Therefore, it is possible that 2 weeks of AMPK loss is not sufficient to reduce muscle cross sectional area; however this requires further investigation. Taken together, these data demonstrate that the modulation of AMPK in MIN mice by fasting and physical activity.

In addition to regulating skeletal muscle mass, AMPK also exerts control over muscle metabolism and mitochondrial quality control (Canto et al., 2010; Laker et al., 2017). In healthy human skeletal muscle, exercise training can improve the fasting induction of AMPK autophagy signaling (Dethlefsen et al., 1985; McConell et al., 2005). AMPK has emerged as a critical regulator of muscle mitochondrial health via its regulation of mitochondrial biogenesis (PGC‐1α) and fission (DRP‐1) (Chen et al., 2019). These processes are essential for skeletal muscle maintenance during periods of energy deficiency, such as fasting and acute exercise. Numerous disease models, including cancer cachexia, have reported that skeletal muscle AMPK is chronically activated, suggesting a state of constant energy stress or deficiency (Bujak et al., 2015; Puppa et al., 2014; White, Puppa, Gao, et al., 2013). Our laboratory has previously reported that severe cancer cachexia activates muscle AMPK signaling in both the Lewis lung carcinoma (LLC) and MIN mouse models of cancer cachexia (Puppa et al., 2014; White et al., 2011). At the same time, PGC‐1α is suppressed (Puppa et al., ,^2012^, ^2014^; White, Puppa, Gao, et al., 2013). In the current study, wheel running did not affect muscle PGC‐1α expression in 12‐h‐fasted MIN mice. The suppression of AMPK phosphorylation did not coincide with the lack of changes to PGC‐1α expression, and differential effects of cachexia on AMPK signaling and PGC‐1α expression have been previously reported (Puppa et al., 2012, 2014; White, Puppa, Gao, et al., 2013).

Our results also confirm that increased physical activity can attenuate the cachexia induction of muscle AMPK and restore suppressed COX activity, which has been previously reported (Hardee, Fix, et al., (1985)). The wheel running increase in muscle COX activity was associated with the level of activity in MIN mice. Also, wheel running induced mitochondrial quality control proteins MFN‐1 and DRP‐1 in MIN mice, which are critical regulators of mitochondrial dynamics. We also demonstrate that the fasting suppression of MFN‐1 and induction of DRP‐1 in MIN skeletal muscle requires AMPK. These results collectively suggest that wheel running is sufficient to restore aberrant AMPK signaling and induce mitochondrial quality control proteins in fasted MIN mice.

While the control of cancer‐induced skeletal muscle protein degradation is widely studied (Hardee et al., 2017; White et al., 2011; White, Puppa, Gao, et al., 2013), there has been a focus on the classical FOXO3a control of E3 ligases, Atrogin‐1, and MuRF‐1, driven by inflammatory signaling (Blackwell et al., 2018; Reed et al., 2012; White et al., 2011). Nevertheless, many different catabolic conditions have investigated skeletal muscle atrophy regulation by metabolic disruptions that induce AMPK signaling (Bujak et al., 2015; Di Magno et al., 2016; Nakashima & Yakabe, 2007; Sanchez, Candau, et al., 2012; Sanchez, Csibi, et al., 2012; Thomson, 2018). Our current data build on these findings by demonstrating that fasting increases FOXO3a activity in MIN skeletal muscle. We establish that increased physical activity in MIN mice can attenuate the induction of FOXO3a by a fast. We also determine that the fasting induction of FOXO3a phosphorylation in MIN skeletal muscle requires AMPK. The loss of AMPK also inhibited MuRF‐1 and Atrogin‐1 in MIN skeletal muscle.

Cachexia is the unintentional loss of muscle mass with or without fat loss that is irreversible with nutritional support alone (Evans et al., 2008; Myers et al., 2019). Roughly 40% of all cancer‐related deaths can be attributed to cancer‐induced cachexia (Argiles et al., 2010). There are currently no approved treatments to prevent or attenuate cachexia's progression (Anderson et al., 2017) and in combination with the high prevalence of cachexia provides a solid rationale for the need to advance our understanding of the regulation of cachexia development. Due to limitations in studying cancer patients, preclinical cancer models have provided valuable mechanistic insight into cachexia regulation. The MIN mouse is an established preclinical model of cancer cachexia that exhibits an overall reduction in cage activity and voluntary wheel activity (Puppa et al., 2014,; Baltgalvis et al., 2010), without reductions in overall food consumption (Narsale et al., 2016). Furthermore, the MIN mouse has decreased gonadal function, and increased inflammation (White et al., 2013b). Cachectic cancer patients consistently demonstrate reduced physical activity (Moses et al., 2004; Nissinen et al., 2018), decreased gonadal function, and increased inflammation; however, cachectic cancer patients often demonstrate reduced food consumption (Evans et al., 2008; Myers et al., 2019). Furthermore, skeletal muscle signaling in the MIN mouse is similar to cachectic cancer patients; however, there are discrepancies which can likely be due to the feeding and activity status of the mouse and human (Fearon et al., 2012). Lastly, the MIN mouse develops intestinal polyps whereas cachectic cancer patients typically have one tumor that can/has metastasized to several tissues. Overall, MIN mice and cachectic cancer patients have many similar characteristics of cachexia, with a few discrepancies that should be considered.

In conclusion, during the initiation of cachexia, the cancer environment's effects on autophagy signaling and mitochondrial quality control protein disruptions are regulated in part by aberrant AMPK signaling. We provide evidence that increased physical activity through wheel running can normalize aberrant fasting‐induced AMPK activation in MIN skeletal muscle. Wheel running suppressed elevated E3 ligase and autophagy signaling. Our results provide evidence that aberrant fasting‐induced AMPK’s regulation occurs in pre‐cachectic MIN mice, and exercise can improve this regulation.

## 4 AUTHOR CONTRIBUTION STATEMENT

D.K.F. and J.A.C. were involved in conception and design of the research; D.K.F. and B.R.C. performed the experiments; D.K.F. analyzed the data; D.K.F., B.R.C., and J.A.C. interpreted the results of experiments; D.K.F. prepared the figures; D.K.F. and J.A.C. drafted the manuscript; D.K.F., B.R.C., A.J.S., M.A.S., H.K., and J.A.C. edited and revised the manuscript; D.K.F., B.R.C., A.J.S., M.A.S., H.K., and J.A.C approved the final version of the manuscript.

## CONFLICT OF INTEREST

The authors have no conflict of interest to disclose.
